# Growth and Division in a Dynamic Protocell Model

**DOI:** 10.3390/life4040837

**Published:** 2014-12-03

**Authors:** Marco Villani, Alessandro Filisetti, Alex Graudenzi, Chiara Damiani, Timoteo Carletti, Roberto Serra

**Affiliations:** 1 Department of Physics, Informatics and Mathematics, University of Modena and Reggio Emilia, v. Campi 213a, 41125 Modena, Italy; E-Mail: marco.villani@unimore.it; 2 Department of Environmental Sciences (DAIS), University Ca’ Foscari, Ca’ Minich, S. Marco 2940, 30124 Venice, Italy; E-Mail: alessandro.filisetti@unive.it; 3 Department of Informatics, Systems and Communication, University of Milan-Bicocca, Viale Sarca, 336, 20126 Milano, Italy; E-Mail: alex.graudenzi@unimib.it; 4 SYSBIO—Centre for Systems Biology, University of Milan-Bicocca, Piazza della Scienza 2, 20126 Milano, Italy; E-Mail: chiara.damiani@unimib.it; 5 Department of Mathematics and Namur Center for Complex Systems—naXys, University of Namur, rue de Bruxelles 61, B-5000 Namur, Belgium; E-Mail: timoteo.carletti@unamur.be; 6 European Centre for Living Technology, University Ca’ Foscari, Ca’ Minich, S. Marco 2940, 30124 Venice, Italy

**Keywords:** protocell, synchronization, collectively replicating chemical systems, dynamical models, artificial chemistries, emergence of novelties, evolvability

## Abstract

In this paper a new model of growing and dividing protocells is described, whose main features are (i) a lipid container that grows according to the composition of the molecular milieu (ii) a set of “genetic memory molecules” (GMMs) that undergo catalytic reactions in the internal aqueous phase and (iii) a set of stochastic kinetic equations for the GMMs. The mass exchange between the external environment and the internal phase is described by simulating a semipermeable membrane and a flow driven by the differences in chemical potentials, thereby avoiding to resort to sometimes misleading simplifications, e.g., that of a flow reactor. Under simple assumptions, it is shown that synchronization takes place between the rate of replication of the GMMs and that of the container, provided that the set of reactions hosts a so-called RAF (Reflexive Autocatalytic, Food-generated) set whose influence on synchronization is hereafter discussed. It is also shown that a slight modification of the basic model that takes into account a rate-limiting term, makes possible the growth of novelties, allowing in such a way suitable evolution: so the model represents an effective basis for understanding the main abstract properties of populations of protocells.

## 1. Introduction 

Protocells are cell-like structures that are simpler than present-day biological cells, but that are nonetheless able (i) to grow by some form of rudimentary metabolism, (ii) to reproduce giving rise to new protocells that are similar to their parents and (iii) to undergo evolution.

While viable protocells do not yet exist, these structures are supposed to have played a key role in the self-organizing processes leading to the emergence of life; moreover their study could be important in order to create new “protolife” forms which are able to adapt and evolve [1]. This endeavor has an obvious theoretical interest, and deep epistemological implications, but it might also lead to an entirely new living technology [2], definitely different from conventional biotechnology.

Different protocell architectures have been proposed [3,4,5,6,7,8,9,10,11,12,13,14,15]. A prominent candidate structure is that of lipid vesicles, that have been observed to fission, under suitable physico-chemical conditions, giving rise to new vesicles [16]. In order to undergo Darwinian evolution, a necessary condition is that the rate of generation of new protocells depends on the chemical composition and physical properties of the parent vesicles. Again, different mechanisms have been proposed [3]; most of them identify a set of molecules that are able to collectively self-replicate, and that affect the growth and fission rates. Once a coupling has been established between the self-replicating molecules (*i.e.*, the “protogenetic material”) and the fission rate, evolution could in principle take place, so it would be appropriate to speak of a true protocell [17].

Theoretical models can be extremely useful to identify the most promising architectures, the most effective ingredients that can lead to their actual buildup [3,18] and to reject improbable hypotheses. Therefore, detailed models of the molecular and supramolecular structures and processes that may be involved are of the utmost importance. On the other hand, it is also interesting to consider the behavior of a protocell as an integrated “individual agent”, using an approach similar to that pioneered by Ganti with his Chemoton model [6]. There is of course a tradeoff, in that such models usually need to resort to a less detailed account of the behavior of their components and therefore to a more abstract and more general description.

We believe that protocell research will benefit from both kinds of approaches, and we show here the outcomes of a new model of the “abstract” kind, that captures some key features of true physical processes. We will consider the case of a container, which can be tentatively identified with a vesicle formed by amphiphilic molecules in water; however, the model is abstract and it can describe also different physico-chemical scenarios. Other molecules, besides those that form the container, may be present in the cell. It is supposed that, when the container reaches a certain size, it becomes unstable and it halves into two approximately equal daughter cells [19].

Note also that being able to synthesize a single protocell capable of fission is not enough; one rather needs a population of protocells capable of sustained increase and fission through successive generations. In order to achieve this goal, the two key processes of membrane growth by means of the uptake of amphiphiles on the surface and that of duplication of the genetic material must synchronize. It is worthwhile to remark that the synchronization is a general requisite not depending on the selected scenario, RNA world, metabolism or whatever. If the genetic material grows faster than the protocell, it would overfill the space, eventually causing the explosion of the membrane. On the contrary, if the protocell grows faster than the genetic material, the latter would be diluted after several divisions (death by dilution). There will be in any case a core group of molecules (or even perhaps a single one) whose duplication must necessarily happen at the same pace as that of the lipid container. We call these molecules (proto-)genetic memory molecules (GMMs).

In previous works [4,5,20] we showed that the synchronization between the replication of the GMMs and the fission of the lipid container is a robust emergent property under a broad set of hypotheses, provided that there is a coupling between the concentration of the GMMs and the rate of generation of membrane molecules. This is indeed the key present-day bottleneck in creating a protocell: there are systems where the vesicle grows thanks to the continuous feeding of lipids from the outside [16], and there are systems where duplication of a set of molecules can be observed [21,22] (the easiest case being that of a single autocatalytic species) but it has so far been impossible to couple them in a single system. For modeling purposes we will assume here that such a coupling actually exists, so the growth rate of the container mass depends upon the concentration of some GMMs. We will also assume that the protocell is turgid, *i.e.*, that it remains spherical, with constant membrane thickness, during growth; we also assume constant lipid concentration in the membrane. This means that during the division and duplication process some material losses should happen, in the case in which the concentration of internal chemicals do not change substantially (a plausible hypothesis in case of fast processes). Under these hypotheses a straightforward geometrical calculation, shown in Section 2.2, allows one to compute the increase in volume of the protocell, given its increase in membrane mass (that is affected by the GMMs).

Different protocell architectures have been proposed, that differ not only for the hypotheses about the chemical make-up of the protocell and of its key processes, but also for their location in the different compartments [1,3,4,5,6,7,8,9,10,11,12,13,14,15,23]. There are models like e.g., the Los Alamos bug [11] where all the key processes (*i.e.*, duplication of GMMs and increase of the container) take place in the lipid membrane or close to its surface. An even more extreme view is taken in so-called Gard models [24], where there is no strict chemical distinction between the two, the lipids also being the “genetic” molecules. We will consider here the most common architecture where the two key processes (formation of GMMs, formation of amphiphiles) are assumed to take place in the internal aqueous phase of the protocell, leaving to future works the discussion of the extension of our results to different architectures.

In this paper the dynamics of the GMMs will be described by resorting to a model, introduced in the 1980s by Kauffman, that has been often used to describe collectively autocatalytic sets of molecules [25]. The various molecular species are assumed to be polymers composed of monomers of various kinds (typically, two), and the reactions are of two types: condensation, where the ends of two polymers join to generate a single one, and cleavage, where a polymer is cut in two parts. It is also assumed that these reactions take place in the internal phase of the cell, only if catalyzed by another polymer, and that polymers are selected to be reaction catalysts at random. In the basic version of the model, the possibility of spontaneous and backwards reactions is ignored.

In the past, we have studied the behavior of this kind of systems using a stochastic approach [26,27], based on the well-known Gillespie algorithm [28]. This choice is motivated by the fact that, in studies on the prebiotic scenarios, it is possible that some molecules are formed at very low concentrations, where resorting to a continuous approach could be misleading. Moreover, we have also explored possible extensions of the basic model (*i.e.*, introducing backward reactions, energy constraints, *etc.* [29,30]). Those initial studies were made in a flow reactor, as it is often done. Surprisingly enough, indeed, the study of self-replication has (almost) always been decoupled from that of protocell dynamics. Self-replication has been considered in a closed vessel or in an open-flow reactor, but none of them area good model for a protocell. Closed systems show major limitations in describing “living” systems (e.g., depletion of reactants, accumulation of wastes), and the way in which a flow reactor is coupled with the environment is very different from that of a vesicle with a semipermeable membrane: the latter allows exchange of some but not all the molecules, while the former intakes all that is included in the input flow, and ejects all the solutes. In a recent paper [31] it was indeed shown that the dynamics of collective self-replication in a vesicle with a semipermeable membrane can be very different from that of a flow reactor [32], and that this property has interesting implications for the study of populations of vesicles. However, in that paper we limited our analysis to a container of fixed size, while in the present work we explore the consequences of coupling the dynamics of the GMMs to that of a vesicle that grows and divides.

We will show in the following that also in this case synchronization can indeed take place, thus allowing sustained growth of a population of protocells, provided that the set of chemical reactions hosts a RAF [33] (Reflexive Autocatalytic Food-generated) set [34], coupled with the growth of the container. A precise definition will be given in Section 3; for the time being, let it suffice to say that its presence guarantees that its molecules are continuously generated [35]. RAF sets can have quite complicated structure, where a fast subpart can lead to the extinction of slower ones; these phenomena will be briefly outlined in Section 4, but it is important to remark that synchronization takes place when a RAF set exists.

Therefore, the model accounts for the sustained growth of a population of protocells. Heredity is also guaranteed by the duplication mechanism, whereby the newborn cells’ compositions are similar to that of the mother one. But in order to assure evolvability, it is also necessary that (i) the population of protocells can be heterogeneous and that (ii) novelties can appear and sometimes spread out. As far as heterogeneity is concerned, it has already been observed in [31] that, if the concentration levels of some molecules are low, e.g. in the micromolar range, and if the protocells are small (say 100 nm), then the internal compositions will be different for simple statistical reasons [36]. Therefore the internal milieu can be different from the bulk, and it can give rise to different growth rates, and therefore to competition at the level of the protocells.

This does not hold for large protocells, whose initial internal compositions should be similar to that of the bulk. In this case, the diversity of the protocell population will be too small. Evolution might be based on the random appearance of new species, that might come from outside, or be generated by non-catalyzed reactions. In this case, in order to ensure evolvability, it is necessary that some new molecules have a chance to survive. As it will be seen, and as it can be intuitively grasped, life for a newcomer can be hard when RAFs are already in place. Indeed, it will be shown that the basic model does not allow these novelties to set in. However, the basic model incorporates some unrealistic assumptions (e.g., instantaneous diffusion), which lead to an exponential growth of RAFs. If these assumptions are relaxed, then it will be shown that novelties can have a finite chance to succeed.

The outline of the paper is the following. In Section 2 we precisely describe the main features of the basic protocell model. In Section 3 we discuss RAF sets and their properties, while in Section 4 we describe and analyze the results of some key simulations. In that section we show that synchronization is achieved if at least a RAF set exists, which is coupled with the container growth, and we analyze the dynamics of several RAFs coexisting in the same protocell. We also speculate on the effects of such competition in a population of protocells. In Section 5 we discuss the appearance of random molecules, and show that they tend to be eliminated in the basic model; as discussed above, we show that at least one modification of the basic model, that takes into account the finite transmembrane diffusion rate of permeating molecules, allows instead the success of some random newcomers. Finally, in Section 6 we will summarize the major outcomes of the study. We believe that not only a new model has been presented, tested and discussed, but that it sheds light on important mechanisms and on generic properties of replicating protocells, that might be valid even beyond the limitations of the present model. We also think that a convincing research agenda can be defined on the basis of these results.

## 2. Model Outline

We here briefly describe the main features of the stochastic model of catalytic reaction network [25], we discuss its behavior in a vesicle [31] and the coupling of the kinetic equations with the growth of the container. Several extensions of the model can be and have been considered (e.g., reverse reactions, spontaneous reactions, *etc.* [29,30]), but for the sake of clarity we will stick here to the basic model.

### 2.1. Entities and Their Interactions

The model describes a system in which simplified chemical species, *i.e.*, monomers and polymers, interact with each other uniquely, via catalyzed reactions. Monomers and polymers are identified by ordered strings of symbols (or “bricks”) from an arbitrary alphabet (e.g., A, B, C …). The length of a polymer is of course the number of its symbols. Each molecular type *i* = 1, …, N is characterized by the string that defines it and by its total number of copies (*i.e.*, total amount) *x_i_,* or by its volume concentration [*x_i_*]. The entire set of number of copies of the different species of the system is denoted by *X* = (*x_1_, x_2_, …, x_N_*).

Only two kinds of reactions are allowed in the system, namely: (i) *cleavage*, the cutting of a species made of more than one brick into two shorter species (e.g., AAAB → A+AAB) and (ii) *condensation*, the concatenation of two species in a longer polymer (e.g., ABA + BA → ABABA). Reactions can occur only if catalyzed by reaction-specific catalysts, so no spontaneous reactions are allowed (by assuming a sufficiently high activation energy for each reaction). Condensation requires an intermediate step with the creation of a temporary complex between one of the substrates and the catalyst. Finally, the presence of backward reactions is also neglected, by assuming a sufficiently high Gibbs energy ∆G for each reaction (catalysts lower both the forward and the backward activation energies, but if the latter is much larger than the former its rate remains very small). Thus, the reaction scheme can be summarized as follows:

- Cleavage: AB + C → A + B + C
- Condensation: (whole reaction: A + B + C → AB + C)
  ○ Complex formation: A + C → A C
  ○ Complex dissociation: A:C → A + C
  ○ Final condensation: A:C + B → AB + C

where A and B are two substrates involved in a specific reaction, C is the specific catalyst for that reaction and A:C represents the transient complex. The kinetic equations can be obtained by applying the law of mass action to this reaction scheme, as detailed in Appendix A.

The overall set of possible reactions in the system defines a so-called “chemistry”. Defining a “chemistry”, essentially amounts to identifying the set of reactions that are allowed and to associate a catalyst to a certain set of reactions (a molecular type can catalyze different reactions, and a reaction can be catalyzed by different molecular types). Following Kauffman [25], catalysts are, in fact, species belonging to *X* and, in particular, each species *i* has a certain (fixed) probability *p_i_* of being the catalyst for a reaction chosen at random (this is of course a strong hypothesis, for a detailed discussion see [17]). In the current version of the model we impose that only species composed of at least three symbols can be catalysts. The allowed reactions are all cleavage reactions, and only the condensation reactions that give rise to species up to a certain maximum allowed length. In the real world natural laws determine the chemistry, in artificial systems like ours each “chemistry” corresponds to a possible artificial world. In Figure 3, Figure 4 and Figure 9 we show some very simple examples of the artificial chemistries we used, but the effects we are showing in this article are general (provided the presence/absence of RAF structures within the involved chemistry) [37].

The dynamics of the system is stochastic and simulated by means of an extension of the Gillespie algorithm [28,38], described in more detail in Appendix A. The reasons why a stochastic approach is necessary in this case are related to the fact that when a new species appears it can be represented by a small number of exemplars, so the effects of randomness may be very relevant [31]. More details on the models we use can be found in Appendix A

### 2.2. The Protocell Model

The dynamic behavior of catalytic reaction networks has been classically investigated in the case of a continuous stirred-tank reactor (CSTR) [26,29,39,40,41,42,43,44,45]. However, a CSTR is very different from a protocell, and in [31] we introduced a more physically sound model, by describing a semi-permeable membrane separating the reaction network from the environment. Yet, the volume of the protocell was assumed to be constant and, accordingly, no association between the dynamics of the reaction network and that of the container growth was present. On the contrary, in this work we investigate the role of the both the variation of the volume and the division process, when the dynamics of the reaction network is associated to the growth of the container.

Similarly to the model described in [31], here we assume a semi-permeable membrane that separates the reaction network from the environment (whose chemical composition is supposed to already be at its equilibrium). The membrane is modeled by allowing only some species to enter and leave the protocell, *i.e.*, those species that are shorter than an arbitrary length *L_perm_*. Conversely, species longer that *L_perm_* remain entrapped inside or outside the protocell. Another key modeling choice is that the concentration of the permeable molecules is assumed to be homogeneous inside and outside the protocell, by hypothesizing infinitely fast diffusion, both in the bulk phases and across the membrane (This latter hypothesis will be relaxed in Section 5 below). In this way the chemical potentials of the permeable species are the same inside and outside. Since the external environment is supposed to be large and constant, the species that can cross the membrane are *buffered*, *i.e.*, their concentrations are constant.

We assume that certain species belonging to *X* are coupled with the growth of the container. These species act as specific catalysts for the production of membrane lipids, assuming abundant and buffered lipid precursors. As for the case of the choice of cleavage and condensation catalysts, the species to be associated to the container growth are chosen randomly with a certain probability.

Let C be a measure of the mass of the container, *i.e.*, the total number of lipid molecules in the membrane (it is proportional to the mass of the membrane under the assumptions made). Then the equation for the growth rate of the container takes the form:

(1) $$\frac{dC}{dt}≅\sum_{i=1}^{N}k_{i}^{cont}\left[x_{i}\right]V_{r}$$

where *V_r_* is the internal volume of the protocell (where reactions occur) and [*x_i_*] is the concentration of catalysts in the internal aqueous phase; the kinetic coefficients *k_i_* are zero for all those species that do not contribute to the container growth. The kinetics of lipid formation are first-order with respect to the concentration of catalyst, because an infinite supply of lipid precursors is considered available inside the protocell; any lipids produced inside the protocell are assumed to go instantaneously to the membrane and not occupy the internal volume.

Protocells can grow and divide: during these processes their form and shape can change [46]. However, although this is an interesting biological fact, we have already shown that it does not affect the synchronization between the internal reactions and the container growth [4], so—for modeling purposes—we can make the useful simplifications that protocells are spherical and turgid with constant membrane thickness.

This hypothesis allows one to determine the relationship between C and the volume, so that Equation (1) provides a rule to determine the volume growth rate. Under the assumptions made, one can show (see Appendix B) that the relation between the amount of lipids and the internal volume of the protocell *V_r_* is equal to:

(2) $$V_{r}\left(C\right)=\frac{1}{6}\pi \delta^{3}{\left(\sqrt{\frac{C}{\rho \pi \delta^{3}}-\frac{1}{3}}\right)}^{3}\underset{\delta \to 0}{\approx }O\left(C^{\frac{3}{2}}\right)$$

As discussed in Section 1, we take here the simplifying approach to the process of cell division by supposing that there is a fixed threshold value of the volume of the protocell, so we assume that when C = θ, the protocell divides into two identical daughters [47]. In the division process the molecules present within the protocell at the division moment will be shared in identical proportion in the two daughters protocells. Under these assumptions, one can show (see Appendix B) that the ratio between the daughter and the mother protocells’ volumes is:

(3) $$\frac{V_{r}\left(\theta /2\right)}{V_{r}\left(\theta \right)}=\frac{\frac{1}{6}\pi \delta^{3}{\left(\sqrt{\frac{\theta /2}{\rho \pi \delta^{3}}-\frac{1}{3}}-1\right)}^{3}}{\frac{1}{6}\pi \delta^{3}{\left(\sqrt{\frac{\theta }{\rho \pi \delta^{3}}-\frac{1}{3}}-1\right)}^{3}}\underset{\delta \to 0}{\approx }{\left(\sqrt{\frac{1}{2}}\right)}^{3}=0.3535\ldots$$

Since the volume of the daughter protocell is approximately $1/\left(2\sqrt{2}\right)$, if the concentration of internal chemicals does not change (a plausible hypothesis), at each duplication around 30% of the internal material will be lost to the external environment. In Section 4.1 we will discuss the disappearance of some not internally produced chemical species during the succession of protocell generations: the material loss just presented increases this tendency but—as explained in detail in Section 4.1—this phenomenon is mainly due to the process of protocell growth and division. The synchronization among internal replicating materials and container indeed is a robust phenomenon, occurring also in duplication processes without material losses [4,5,20].

## 3. Collective Autocatalysis and RAF Sets

Different researchers have suggested several interesting models [17,24,25,39,40,41,42,48,49,50,51,52] that—besides their differences—all share the abstract idea that “collectively replicating chemical systems” can support both genetic and metabolic functions. In order for a protocell to duplicate, it is necessary that the molecules grow in number; very often (both in models and in present-day living beings) this growth is based upon the catalytic activities of some of the involved species, thus giving birth to the so-called “collectively autocatalytic reaction sets”. Identification of such collectively autocatalytic sets is therefore a major issue in studying these systems.

**Figure 1:**
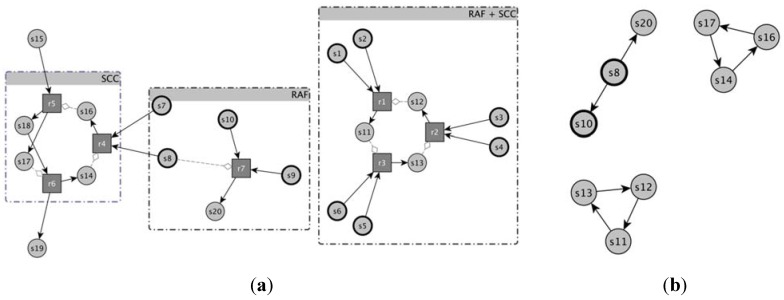
An example of representation of a catalytic reaction network (a) by means of a complete reaction graph and (b) the same example by means of the catalyst → product representation. (**a**) Circles stand for molecular species and in particular bold circles represent species belonging to the food set F. Squares depict reactions, straight arrows indicate the participation of a species to the reaction as substrate (edge points to the reaction) or product (edge starts from the reaction). Catalysts are represented by gray lines ending with diamonds. For the sake of comprehension, both molecular species and reactions forming RAFs and SCCs are grouped together; (**b**) In this representation just the catalytic activity of the reaction network is depicted, and no information about the substrates of the reactions is present. By means of this representation two SCCs are clearly visible and they are formed from s11, s12, s13 and s14, s16 and s17, respectively.

A nice way of identifying the sets of chemical species able to catalyze their own growth is that of using a digraph representation [53] (“complete representation” in the following—see Figure 1) where circles stand for molecular species and squares depict reactions; species can participate to reactions as substrates (continuous links pointing to reactions in Figure 1) or products (continuous links starting from the reactions), or as catalysts (dotted links pointing to reactions in Figure 1). Another useful representation, besides this complete picture, is the simple directed graph (“catalyst-product graph” in the following), where there is an edge from a molecular species to all those species whose production reactions are catalyzed by that species. By means of this last scheme, the identification of subparts (subgraphs) potentially able to collectively replicate is a relatively simple task: these subparts are composed of the so-called Strongly Connected Components (or SCC), subgraphs where it is possible to reach every single node starting from any other node. This means that the presence of only one chemical species (one node) can enable the production of other species (other nodes), that in turn can enable the production of other species, until all species belonging to SCC are produced.

This structure is therefore a possible component of a dynamic engine able to grow and reproduce [26,29,30]. However, in order to grow SCCs need the presence of the substrates required for their construction: so, real self-reproducing objects should be searched on the more complete digraph where reactions, substrates, products and catalysts are all represented. In this digraph these structures are called RAFs (Reflexively Autocatalytic, Food generated sets), where the stress on the F (food) part highlights the requirement that the continuous presence of at least a subset of the chemical species is necessary and required [53,54] (see note [55]). In the model described here, the food molecules are supplied from outside: therefore they must be present in the external environment and be able to cross the membrane.

A RAF is the union of all the reactions allowing the complete self-reproduction of the involved chemical species, so it may be composed of several separate subparts each one able to separately self-replicate: if these subparts cannot be further decomposed they are called irreducible RAFs (irrRAFs) [54]. IrrRAFs therefore are composed of SCC (in the catalyst-product graph) able to catalyze the production of their own substrates (if not already available) or linear chains of reactions whose root is catalyzed by some chemical species whose presence is guaranteed (the “Food” of the RAF sets). These structures can constitute the root of other linear chains of reactions, whose existence depends on these objects [17]; note that also these “tentacles” are included in the irrRAF set.

A very interesting and much studied case is that of random reaction systems, where the molecules are generated at random as well as their chemistry; in this case, major questions concern the possibility of observing collectively autocatalytic systems, *i.e.*, RAF sets, or strongly connected components, *i.e.*, SCCs. Indeed, both depend upon the probability p that a molecule chosen at random catalyzes a reaction chosen at random: if this probability is very low, neither are observed, while, if it is high, the system is full of both. Apart from these trivial regimes, there is an interesting region of intermediate values. It has been observed [31] that the probability of observing SCCs becomes non-vanishing for smaller values than those of observing RAF sets. Therefore there exists a region of p values where SCCs are found in the catalyst-product graph but, since there are no RAFs, they are not continuously supplied with the necessary substrates and are therefore unable to sustain a continuous growth of the number of their molecules. This phenomenon had been described before as a “fragility” of the cycles that are observed in the catalyst-product representation [26,45].

## 4. Synchronization

As anticipated in Section 1, in order to achieve a viable protocell evolution, the two key processes of duplication of the internal material and of membrane growth by means of the uptake of amphiphiles on the surface must take place at the same rate, *i.e.*, they must synchronize [56]. It is worthwhile to remark that the synchronization is a general requisite not depending on the selected scenario, RNA world, metabolism first or whatever. If the internal material grows faster than the “container”, it would overfill the space. On the contrary, if the container grows faster than the internal material, the latter would be diluted after several divisions. In previous works [4,5,20] we have shown that the synchronization between the aforementioned processes is a robust asymptotic emergent property, which is approached in successive generations.

If the kinetic equations for the GMMs are linear, and if some of them are linearly coupled to the growth of the container, then the model can be tackled with analytical methods. Synchronization can be analytically proven and, in the case where the kinetic coefficients are non-negative, the asymptotic cell division time can be also analytically computed as well as the total amount of molecules close to the division point [4,5,20]. Some nonlinear cases can also be shown to be amenable to an analytical approach, moreover several numerical investigation of nonlinear interactions show that synchronization is indeed a robust property, that holds under various hypotheses about the kinetic equations and also under various hypotheses about the protocell architecture. It is interesting to observe that synchronization can be achieved even in the case of chaotic equations for the GMMs, and that it turns out to be robust also when stochastic division is considered. We refer the interested reader for more details and for a thorough discussion to [4,5,20,57], while we limit ourselves here to observe that synchronization, although widespread, cannot be taken for granted, but that it has to be verified on the different kinds of dynamical equations. It is therefore interesting to ascertain under which conditions the model described in Section 2 can show synchronization.

### 4.1. Synchronization and the Role of RAF Sets

Let us observe that the model has no intrinsic distinction between catalysts and substrates, so the same molecular type can play either role in different reactions; if it is consumed as a substrate in a fast reaction, the system will be depleted of that type, and the reactions that need it as a catalyst will be slowed down and eventually stopped. Note also that molecular types that are neither produced nor consumed by catalyzed reactions will have, just before division, the same number of exemplars as they had in the beginning, so their concentration will be reduced until eventually a protocell with a single copy of that species will appear. At division time, only one of the daughter cells will inherit that copy, the other one having none: thus this event is the starting point of a protocell lineage without this particular species.

Therefore, we arrive to the conclusion that only those molecular species that are produced by the system reactions have a chance to survive the division process. We will not consider below some cases, where synchronization occurs in quite “trivial” ways; for example, we will not consider the case where a molecular type in the food set directly contributes to the growth of the container. Other similarly trivial cases will be ignored, such as the case where A and AB are part of the food, and (i) another food molecule catalyzes their condensation to AAB; (ii) AAB is not the substrate of any reaction in the given chemistry; and (iii) AAB contributes to the container growth. In this case continuous production of a molecule coupled to the container is assured. These cases can be tackled with the technique used in our present works and they lead to synchronization. We will also suppose below that no food molecule is also a catalyst, therefore RAF sets need to include a SCC.

**Figure 2:**
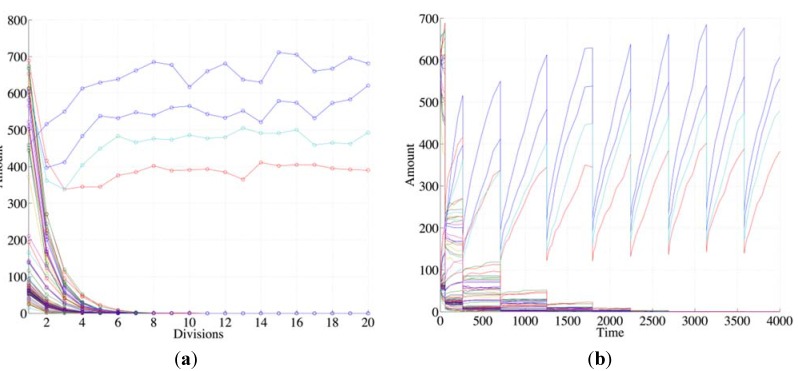
The plots show the surviving of the chemical belonging to a RAF set (the RAF structure here used is the same of Figure 4b, left schema) with respect to other chemical species not belonging to a RAF. (**a**) The amount of each molecular species close to the division time (note that the irregularities beyond the 10th division are due to stochastic effects); (**b**) the same as before, with the addition of the protocell internal concentration levels between each divisions (first 9 divisions).

Species can be generated by several reactions but, as it has been observed in previous works, collective autocatalysis is fragile unless a RAF set is present. Therefore, it is reasonable to guess that the presence of a RAF set, coupled with the growth of the container, is a necessary condition for robust synchronization. This conjecture has been tested and confirmed in many different simulations, like the one shown in Figure 2, where one can see that species not belonging to any RAF (“non RAF species”) undergo dilution while those belonging to the RAF survive and undergo synchronization in successive generations (Figure 2, as all following figures, shows typical behaviors that do not depend on the details of the particular artificial chemistry used).

Suppose now that the protocell hosts a single irrRAF: if there were no cell divisions (and no resource limitations), its growth would be exponential [58]. The synchronized cell division leads to an approximately constant number of molecules at the end of each growth period, but the misleadingly “quiet” appearance of Figure 2 indeed hides the fact that at each division the number of protocells doubles and so do the quantities of chemicals. This exponential growth may be an approximately correct description of the phenomena on a limited time scale, but rapidly this fast increase leads to a condition where some nonlinear phenomena (e.g., resource limitation) become important: in this situation the dynamics of growth changes, leading to significant effects on the protocells themselves, as we shall see in Section 5.

**Figure 3:**
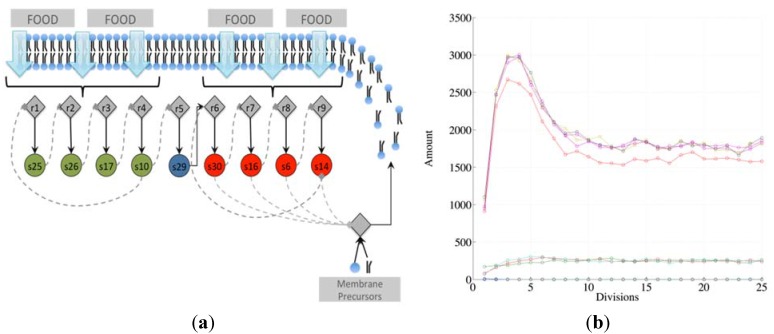
(**a**) The schematic representation of a system containing a unique RAF composed of two distinct, strongly connected components (for reason of simplicity only the reactions belonging to the RAF are shown). The SCC composed of the species 25, 26, 17, and 10 (green circles) produces the substrate needed (species 29) for the closure of the second SCC composed of species 30, 16, 6 and 14. The second SCC (in red) is coupled with the container growth; (**b**) The evolution of a protocell lineage with this irrRAF along 25 division processes. On the x-axis the division is represented while on the y-axis the amount of molecular species at the division time are shown (depending upon the parameters used in this case, the species belonging to the first SCC have concentration levels higher than species belonging to the second SCC).

Note that there exist different kinds of irrRAFs. An interesting case, shown in Figure 3, is that of a protocell hosting a single irrRAF that can be considered as “heterogeneous”, as it is composed of two coupled SCCs, one of them being coupled also to the growth of the container. As expected, synchronization is observed also in this case.

Moreover, a single protocell may host different irrRAFs. In the case of independent (*i.e.*, non-directly coupled) irrRAFs in the same protocell, it has been observed (and it can be easily understood) that if different irrRAFs have the same growth rate, then they all survive, even if they have different coupling coefficients with the container, or even if some of them are not coupled at all. In this case they are like parasites of the irrRAFs that contribute to the container growth.

However, having exactly the same kinetic parameters may be an unusual situation. Suppose for example that there are two irrRAFs, and that the fast one is coupled with the container growth while the other is not. In this case one observes the dilution of the slow one, while the other synchronizes with the cell duplication. There are however some strange phenomena that may be observed when an irrRAF is “dying”, and they will be discussed below. The same behavior (*i.e.*, dominance of the fastest) is observed when there are more than two irrRAFs growing at different rates, all coupled to the growth of the container.

A dual case is that of two irrRAFs, where only the slowest one is coupled to the growth of the container while the fast one is not. In this case the molecules of fastest irrRAFs increase their concentrations continuously in the protocell at division time, so this might lead to a breakup of the protocell. This is in any case an event outside the scope of the presented models. Also this behavior can be generalized to more than two irrRAFs.

Note also the intensity of the coupling among irrRAFs and the membrane has a major role: very high (but physically implausible) coupling values can change some of the previous observations. Indeed, the faster the container growth rate, the smaller the number of molecules at each division: for example, also in the case of irrRAFs having equal growth rate, once the species concentrations approach very low values at division time all the molecules belonging to one irrRAF could fall into just one of the daughters, leading to the birth of protocells without its presence.

### 4.2. The Fate of Declining irrRAFs

It is worthwhile to examine the fate of those irrRAFs that tend to be eliminated due to the presence of other, faster ones that are coupled with the growth of the container: in this case we can observe interesting phenomena due to the role of stochasticity when just one or a few exemplars of a given molecular species survives. Note that, while it is possible to rule out some phenomena related to a physically implausibly high contribution of a single or a few molecules to the growth of the container (as discussed at the end of the previous section) it is impossible to simply rule out as irrelevant the case in which just one copy of GMM survives. Indeed, even in present-day evolved cells, e.g. bacteria, there are genes that are present in single copies.

**Figure 4:**
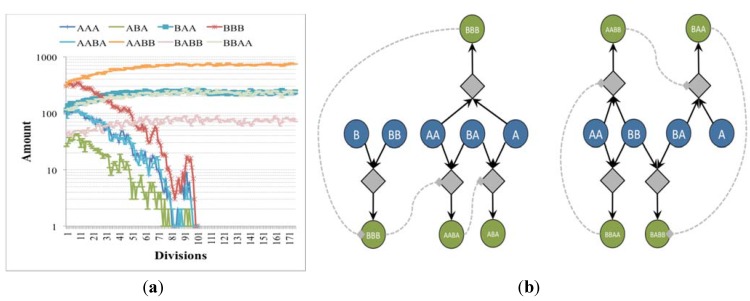
(**a**) The number of molecules of chemical species close to the division time of (**b**) two irrRAFs having different growth rate (blue circles indicates buffered species, green circles free species, diamonds the reactions). In this case the limitation of the slower irrRAF is due to the low concentrations of the substrates of one of its reactions.

Let us consider again the case of two independent irrRAFs having different growth rates, no matter the intensity of the coupling with the membrane: the concentration of species belonging to the slowest one slowly decreases, reaching so low numbers of molecules that stochastic effects start to play a major role (Figure 4). It should be noticed that, as long as an irrRAF in a protocell has at least one molecule of one of its member species, it has the possibility of replicating the other species and of restarting its growth. The only way to remove an irrRAF is that of removing from the protocell all the molecules of all of its species: so the higher the number of species belonging to the irrRAF, the more difficult its removal. Therefore once an irrRAF has reached very low concentration levels its removal may require very long times, but eventually it is likely to occur (remember that the model is stochastic, so here we have a stochastic process with an absorbing boundary at zero concentration).

Since this process requires some generations to show up, in order to understand what may happen it is necessary to consider a population of protocells. Due to the stochastic character of the removal of the slow irrRAF, it is highly unlikely that it takes place at the same time in all the protocells. Therefore, after some time, there will be some protocells with two irrRAFs and some with only one, *i.e.*, the fast one (ignoring the possibility that new irrRAFs are generated).

Let us suppose that at a certain time point t there are just two kinds of vesicles: those that have both irrRAFs and those that have only one, and let Y_t_ and X_t_ respectively be their numbers. The fast irrRAF is present in both population, and it synchronizes with the container, so the evolution time can be described by a discrete map with constant Δt across the various generations. In the division process described in Section 2 it has been observed that, since the total volume of the two daughter cells is less than that of the parent one, some internal molecules are lost at each duplication; however we suppose that the numbers of copies of the molecules of the fast irrRAF are high enough, so that the probability that a protocell appears without any RAF, because both have been lost, is negligible (the reasoning below could be modified to take also a small loss term into account).

For the sake of simplicity, we can suppose that each of the N species belonging to the slow irrRAF has only one molecule: so, the probability α of giving birth to a protocell with only one irrRAF is constant; moreover, for the time being, we will suppose that the probability of losing all the molecules of the slow irrRAF in the lost volume at division process is also negligible. Under these assumptions, α = 1/2^N^. If the damaged but not empty irrRAFs are able to recover one copy of all their molecules, then after duplication a population of M protocells, which have initially had both irrRAFs, will be composed of M + (1−α)·M protocells owning both irrRAFs (we neglect the chance that both irrRAFs are eliminated in the lost volume in duplication, that is much smaller than the chance that only one is), and by αM protocells owning a single irrRAFs. Note that the duplications of protocells having only one irrRAF surely gives birth to two protocells having only one irrRAF, and that this population may be increased by the one-irrRAF offspring of the other one.

**Figure 5:**
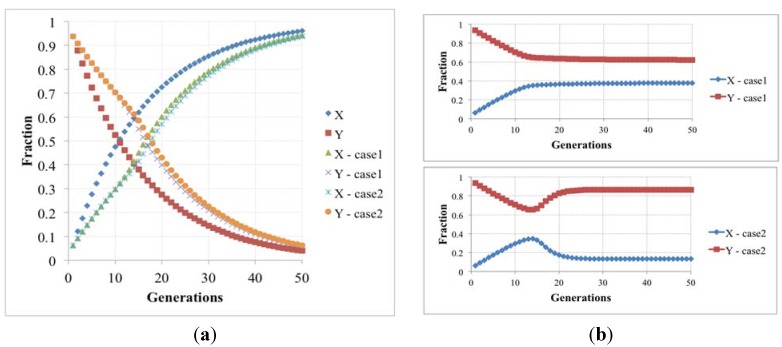
(**a**) The fraction of protocells with only one (X) and two (Y) irrRAFs having different growth rates, in the case of no limitations, and resource limitation or overcrowding (we show two cases with different β values, respectively 5.0 × 10^−6^ and 1.0× 10^−6^); (**b**) The latter case when the losing irrRAF has positive effects on the resource limitation or overcrowding (case1 and case2, where β changes from 5.0 × 10^−6^ to 5.88 × 10^−6^ and from 1.0 × 10^−6^ to 1.5 × 10^−6^, respectively).

So it is possible to define the simple iteration map:

(4) $$\left\{ \begin{array}{l} X_{t+1}=2X_{t}+\alpha Y_{t}\\ Y_{t+1}=\left(2-\alpha \right)Y_{t} \end{array}\right.$$

that describes the time change of the two protocell subpopulations: both actually increase, as is typical of linear systems, but the '’s rate is higher than that of the Y’s, so the ratio of Y’s to X’s vanishes (see Figure 5). Under more realistic conditions, where nonlinear growth limiting terms do appear, this would imply extinctions of the Y’s: within the protocell population the fraction of protocells having both RAFs eventually vanishes, leaving only those with the fast one.

But this result may be a consequence of the linearity of Equation (4). One can introduce growth-limiting terms, due to e.g., overcrowding or resource limitations, like those of Equation (5), that are frequently used in population dynamics; however, also in this the fraction of protocells having both RAFs eventually vanishes, like in the previous case.

(5) $$\left\{ \begin{array}{l} X_{t+1}=2X_{t}+\alpha Y_{t}-\beta \left(X_{t}+Y_{t}\right)X_{t}\\ Y_{t+1}=\left(2-\alpha \right)Y_{t}-\beta \left(X_{t}+Y_{t}\right)Y_{t} \end{array}\right.$$

A different behavior can be observed if the β parameter (that rules the growth-limiting term) takes different values for X and Y populations: if the presence of the losing irrRAF provides a positive contribution to the protocell dynamics, for example by giving increased resistance to overcrowding or improvement of the resource exploitation, then the fraction of protocells having this irrRAF does not vanish, and the two can coexist in the asymptotic population. This case constitutes an interesting example of interaction of spontaneous processes occurring at different levels of organization.

## 5. Novelties

There has been a lively discussion in the literature about the emergence of novelties in living systems (please refer to [17,59]). In the models presented in this paper, novelties could be identified with the unexpected appearance of few molecules of one or more chemicals species, due to spontaneous reactions (that may be slow but not completely absent) or to intake from the environment [60]. A major issue concerns the evolvability of these models: if every new molecule would always be eliminated, then the possibility to undergo radical changes would be limited by the chosen “chemistry”. The issue is far from obvious: as we have seen, exponential growth of some molecular types can take place in the protocell, and newcomers might easily be eliminated in the “survival of the fittest” competition (as discussed in Section 4 with reference to competition among RAFs). The fastest process is the only one surviving, whereas the slower ones activities have to vanish. Therefore, in the original model with exponential growth a “truly new” molecule (*i.e.*, one that has not and cannot be generated by the existing chemistry, given the initial conditions) could survive and replicate only if it were able to interact with an existing RAF, for example being recruited by it [61].

However, as has already been proposed in Section 4.2, exponential growth can be a first approximation to more realistic models with limitations to growth. Let us consider for example one of these possible rate-limiting steps, *i.e.,* the finite diffusion rate of chemicals through the cell membrane. In the following part of this section, we will consider a model that differs from that of Section 2 in that the chemical equilibrium of the permeable species through the membrane is not instantaneous, but is ruled by Fick’s law [62] (while the impermeable species cannot cross the membrane, as in the case of Section 2). Therefore:

(6) $$\frac{dM_{i}}{dt}=D_{i}A\left(\left[M_{i}^{out}\right]-\left[M_{i}^{in}\right]\right)$$

where dM_i_/dt is the rate of intake of the chemical *i* (number of molecules), D_i_ is proportional to its diffusion coefficient divided by the (constant) membrane thickness; A is the surface of the protocell and [M_i_^out^] and [M_i_^int^] are the concentrations of the chemical *i* outside and inside the protocell, respectively.

**Figure 6:**
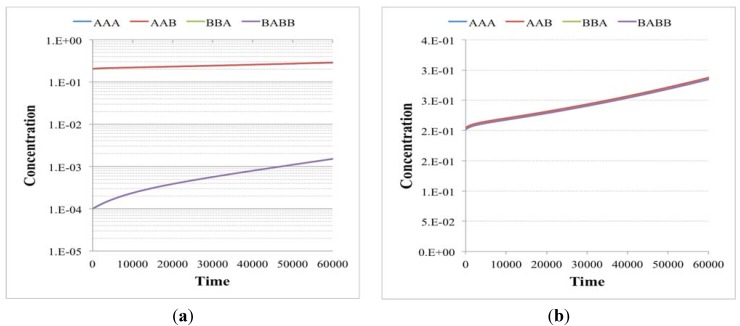
Concentrations in time (arbitrary units) of chemical species of independent irrRAFs having the same growth rate but starting from different initial concentrations (**a**) when the second irrRAF makes its first appearance (plot from the birth of the protocell to its duplication event—note the logarithmic scale on the y-axis) and (**b**) when the second irrRAF reaches the first one, at the 20th generation. Simulations made with irrRAFs competing for same substrates gave similar results.

**Figure 7:**
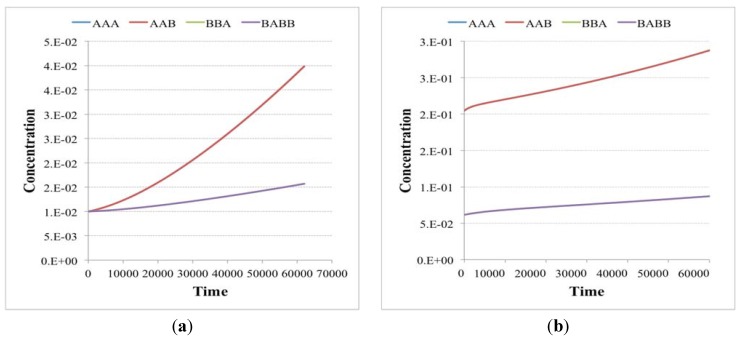
Concentrations in time (arbitrary units) of chemical species of independent irrRAFs having the different growth rate (**a**) when the second irrRAF makes its first appearance (plot from the birth of the protocell to its duplication event) and (**b**) when the second irrRAF synchronizes with the first one, at the 20th generation. Simulations made with irrRAFs competing for same substrates gave similar results.

**Figure 8:**
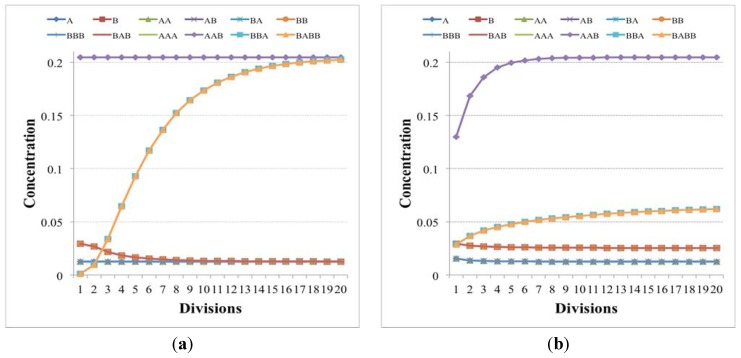
Concentrations (arbitrary units) of chemical species close to the division time in the case of (**a**) two independent irrRAFs having the same growth rate but starting from different initial concentrations and (**b**) two independent irrRAFs having different growth rates. Simulations made with irrRAFs competing for same substrates gave similar results.

**Figure 9:**
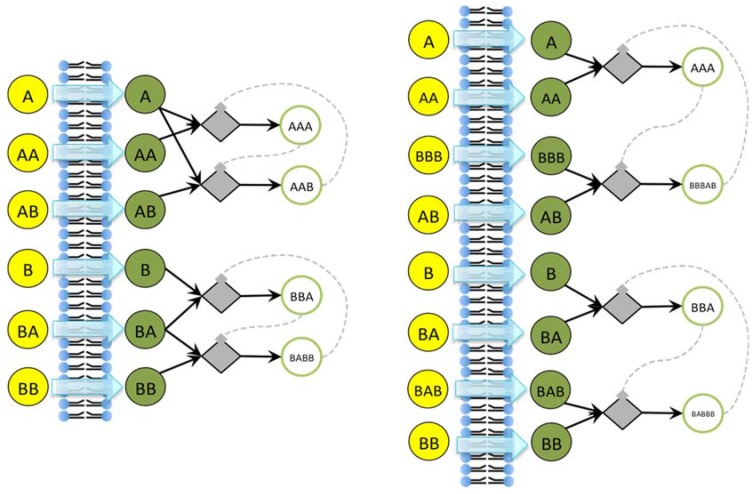
The irrRAFs schema of the two examples presented in the text. Yellow and green circles are the substances that can pass through the membrane (the concentrations of the yellow ones being keep fixed during the simulations), whereas diamonds and white circles represent, respectively, the reaction and the reaction products. Reactions indeed occur on both sides of the membrane, but because the different size of protocell and environment their effects are significant only on the interior of the protocell, while on the other side they are quickly (in our model, instantaneously) diluted—see Section 6 for a discussion

In this case, irrRAFs having growth rates equal to that of the already present ones but starting from very low concentrations have the possibility of increasing their presence and reach the already present irrRAFs (Figure 6 and Figure 8a). Moreover, depending on the relative substrate intakes, irrRAFs having different growth rates can coexist (Figure 7 and Figure 8b). While the simulations refer to the RAFs of Figure 9, their general properties are shared by several other cases that have been examined and therefore provide some indications on how advantageous (or at least not too disadvantaged) true novelties can indeed develop within the system.

## 6. Conclusions: Remarks and Indications for Further Works

We believe that the model presented here could provide a convincing picture, at an abstract level, of the main features of the relevant processes involved in protocell growth and division. The model can be improved in different ways, some of which will be summarized below, but it can also be used in its present version to address some important issues in protocell research.

First of all, let us recall that a population of units of evolution is “any population of entities with the properties of multiplication (one entity can give rise to many), variation (entities are not all alike, and some kinds are more likely to survive and multiply than others), and heredity (like begets like)” [63]. The protocells considered here do indeed have these properties: multiplication and heredity are fairly obvious and they are “built in” the model, while the necessary heterogeneity of protocells can be provided by some randomness at division time (not explicitly described here) and/or also by the smallness of the protocell itself (individual protocells, if small enough, can host different subsets of the complete set of chemicals). It has been observed [31] that a small vesicle can host on average very few exemplars of a given molecular species (e.g., just one in a 100 nm vesicle, if the concentration is 10 μM; if the concentration is 1 μM then there will be just an exemplar of that type in ten protocells).

It is interesting to observe that the results shown above prove that not only protocells can be different, but that they may also host new types of molecules (*i.e.*, those that cannot be generated by the catalysts that are included in the “chemistry”, and that may come from outside or from rare spontaneous reactions) and make them grow—if the growth limiting terms are taken into account as it has been proposed in Section 5. Therefore a population of this kind of protocells has the features required to be truly evolvable [17,63].

The previous observation on small protocells opens an interesting question: why are there protocells at all? They tend to be taken for granted, or to be given some superficial explanations. But, if the key reactions all take place in the internal water phase, what makes a protocell different from a similarly small portion of the bulk? The latter would contain, on average, the same substances at the same concentrations as the protocell. Confinement of the reaction products and substrates would not be an answer, since the concentrations are the same.

There are at least two different (and not conflicting) possible answers to the question concerning the necessity of compartments: (a) the smallness of the vesicle; or (b) a possible active role of the membrane. The first one has already been discussed above: small vesicles can have an internal composition that is very different, not only from that of other protocells (thus ensuring heterogeneity), but also from that of the bulk, thus allowing different reaction pathways to dominate their internal environments.

As far as alternative (b) is concerned, it is indeed highly plausible that the lipid membrane creates a local environment close to its boundaries that is markedly different from the bulk and that these surface effects modify the chemistry, by favoring some reactions and inhibiting others. In this case the membrane would itself act like a kind of catalyst, and would create a local environment different from the bulk. This would happen on both sides, but here the advantage of a closed vesicle is apparent: the molecules generated inside it will be trapped (if non permeable) in a small volume, so their concentrations will increase, while those that are generated on the outer side will freely diffuse in the bulk, without significantly affecting the external concentrations (at least until there are not very many protocells). It has also been shown that some superconcentration phenomena can appear in these cases [64]. The active role of the membrane has been advocated in models, like the Los Alamos Bug or GARD, where the key reactions take place in the membrane itself; the above remarks show that it can have a major active role also in the case where the key reactions take place in the water phase, but are affected by surface phenomena.

Finally, note that the presence of high protein concentrations in small vesicles are also experimentally reported, as for example in [65]; these differences between internal and external chemical concentrations could induce the presence of interesting osmotic processes [66].

As far as model improvements are concerned, there are of course many important directions. Indeed, we think that the picture that has been outlined above represents a rich class of models and opens up an interesting agenda for further research.

A major topic concerns the robustness of our analysis (concerning synchronization, role of different irrRAFs, novelties) in different protocell architectures, like the Los Alamos Bug or the GARD models. Previous works on synchronization with different equations for the replicators makes us confident that the results can actually be robust, but this has of course to be proven.

The appearance of novelties represents a major research topic: it would be important to better determine under which conditions the novelties are actually likely to survive and spread.

The model of the reactions could also be significantly improved, e.g. by considering a structure-dependent catalytic activity, instead of using a uniform probability of catalysis *p*. Other improvements (that have already been considered in previous works on flow reactors) involve considerations of backward reactions and the introduction of energy constraints.

Let us recall, from the above discussion, that there are at least two different (although not mutually exclusive) explanations of the importance of closed containers: heterogeneity due to smallness, and an active role of the membrane. Both should be explored with appropriate versions of the model in order to understand their features.

Other improvements are of course also possible. The model presented here can be the basis for a set of explorations of different alternatives, and it can be useful to cast basic questions about protocells in a precise form, amenable to analysis and simulation.

## Figures and Tables

**Figure B1:**
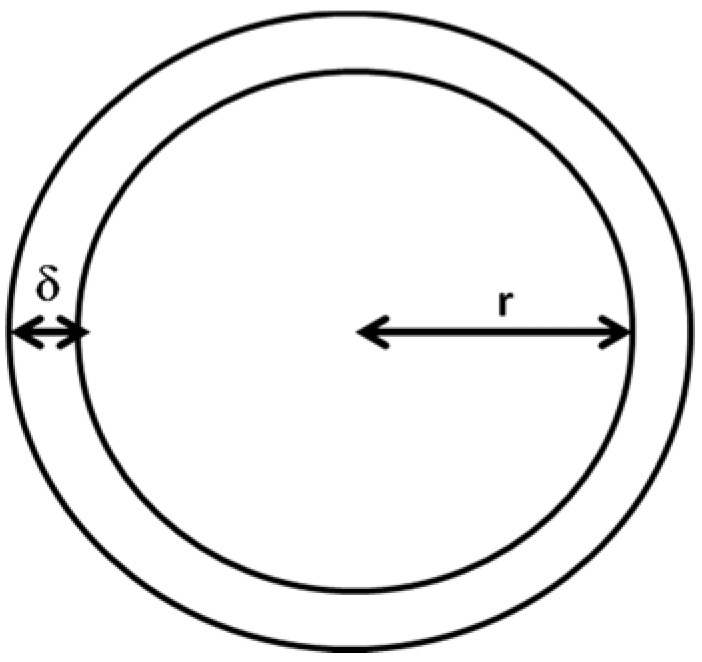
The schema of a protocell with a membrane of constant thickness.

## Appendix A

The systems described in this article require the modeling of two main interacting subsystems: (i) the chemical reactions dynamics happening inside the container and (ii) the container itself.

For simplicity we suppose that the internal environment is perfectly mixed, the exchange with the external environment being ruled by the container properties (in some of our previous works we suppose a continuous inflow water with constant concentration of solutes—a CSTR system—whereas in this article we suppose the presence of a semipermeable membrane). For efficiency purposes, the container exchange properties are modeled by means of ODEs, whereas within the internal physical space the dynamics of the chemical reactions (ruled by the mass action law) is modeled by means of two different schema.

The first schema consists of the integration of the differential equations describing the system. For each cleavage *r* the concentration change of the chemical *x_k_* that breaks onto chemicals *x_i_* and *x_j_* because the presence of the catalyst *x_c_* is:

(A1) $$\frac{d\left[x_{i}\right]}{dt}=\frac{d\left[x_{j}\right]}{dt}=-\frac{d\left[x_{k}\right]}{dt}=Kcl_{r}\left[X_{c}\right]\left[X_{k}\right]$$

where *Kcl_r_* is the kinetic constant of the reaction *r*.

Similarly, for each condensation *d* (realized by means of the two-step process described in Section 2.1) the concentration change of the chemicals *x_a_* and *x_b_* that condensate in chemical *x_p_* because the presence of the catalyzer *x_c_*, through the intermediate reactions involving the temporary chemical complex *C_i_* is:

(A2) $$\left\{ \begin{array}{l} \frac{d\left[C_{i}\right]}{dt}=Kcf_{d}\left[x_{a}\right]\left[x_{c}\right]-Kcd_{d}\left[C_{i}\right]-Kfc_{d}\left[C_{i}\right]\left[x_{b}\right]\\ \frac{d\left[x_{a}\right]}{dt}=Kcd_{d}\left[C_{i}\right]-Kcf_{d}\left[x_{a}\right]\left[x_{c}\right]\\ \frac{d\left[x_{b}\right]}{dt}=-Kfc_{d}\left[C_{i}\right]\left[x_{b}\right]\\ \frac{d\left[x_{c}\right]}{dt}=Kcd_{d}\left[C_{i}\right]-Kcf_{d}\left[x_{a}\right]\left[x_{c}\right]+Kfc_{d}\left[C_{i}\right]\left[x_{b}\right]\\ \frac{d\left[x_{p}\right]}{dt}=Kfc_{d}\left[C_{i}\right]\left[x_{b}\right] \end{array}\right.$$

*Kcf_d_*, *Kcd_d_* and *Kfc_d_* are the kinetic constants of the complex formation, complex dissociation and complex condensation with the chemical *x_b_*.

Each chemical can be substrate or product of none, one or more reactions (depending on the chosen artificial chemistry), its overall concentration change being the sum of all concentration changes caused in each reaction in which the chemical is involved.

In this case the integration with the container (CSTR or permeable membrane) is straightforward. This schema is very effective in case of relatively high chemical concentrations; on the other hand it present difficulties when the concentrations are very low and the granularity of the system becomes significant.

So, the second schema directly simulates the disappearance and the formation of each single molecule by means of a Gillespie algorithm [28,38], and replicates the reaction schema of Section 2.1. The Gillespie reaction constants can be derived from the kinetic constant by means of the relations:

(A3) $$\left\{ \begin{array}{l} Ccl_{r}=\frac{Kcl_{r}}{V}\\ Ccf_{d}=\frac{Kcf_{d}}{V}\\ Ccd_{d}=Kcd_{d}\\ Cfc_{d}=\frac{Kfc_{d}}{V} \end{array}\right.$$

In this case the integration with the container module (modeled by means of differential equation) is less obvious; nevertheless, the integration of these equations by means of the Euler schema can allow a very simple integration. Being a first order method, this schema implies variable changes directly linked to a finite time interval: in our framework this time interval is directly derived from the Gillespie framework, which therefore becomes the main systems’ engine. So the general schema is:

- While (simulation is non ended)
  (a) The Gillespie framework holds, and computes a finite time interval Δ*t_g_*.
  (b) The simulation time is adjourned *t_s_* = *t_s_* + Δ*t_g_*.
  (c) For each chemical *x_i_*:
    (i) The time passed from the chemical last change is adjourned *LC_i _* = *LC_i_* + Δ*t_g_*.
    (ii) Its flows through the container is adjourned *fw_i_* = *fw_i_* + f(*rules*, *LC_i_*).
    (iii) If |int(*fw_i_*)-*fw_i_*| ≥ 1, the number of molecules of *x_i_* is varied of int(*fw_i_*), *fw_i_* is decremented by int(*fw_i_*) and *LC_i_* is set to 0.

Int(*X*) and f(*rules*, *LC_i_*) indicate respectively the not fractional part of X and the dynamical rules (derived from of the differential equations description) that drive the chemical *i* mass exchange with the external environment. The time interval Δ*t_g_* is very tiny (it correspond to the formation/disappearance of few molecules) and therefore is compatible with the Euler schema.

For simplicity reasons, if not differently indicated in this article the reactions’ kinetic constants are set to *Kcl_r_* = 10 (∀*r*), *Kcf_d_* = 5000 (∀*d*), *Kcd_d_* = 250 (∀*d*), *Kfc_d_* = 500 (∀*d*) (arbitrary units); note, however, that the structure of the relationships among RAFs, membranes and protocell growth does not depend on the particular values of these parameters (whereas, of course, these parameters determine the rates of the phenomena).

By the way, note that if the volume is time dependent (V = V(t)) and if the process is quasi-static (the volume variation is not too fast) the relations in A3 can allow the Gillespie simulation of systems where the container volume can vary in time (it is enough to take into consideration the volume changes and recalculate the Gillespie reaction stochastic constants during the simulation). See also [27] for further details.

## Appendix B

The meaning of various terms and the hypotheses are described in Section 2.2.

Let the internal volume where all the relevant reactions occur (*i.e.*, the Gillespie volume) be $V_{r}=\frac{4}{3}\pi r^{3}$ , and let the external volume of the protocell be $V_{e}=\frac{4}{3}\pi {\left(r+\delta \right)}^{3}$ , where δ is the thickness of the membrane; accordingly, the spherical shell delimited $V_{m}=V_{e}-V_{r}=\frac{4}{3}\pi {\left(r+\delta \right)}^{3}-\frac{4}{3}\pi r^{3}$ by the internal and the external spheres allows to determine the membrane volume as follows:.

Assuming a constant concentration of lipids:

(B1) $$\rho =\frac{C}{V_{m}}=\frac{C}{\frac{4}{3}\pi {\left(r+\delta \right)}^{3}-\frac{4}{3}\pi r^{3}}$$

From Equation (B1) we can derive that:

(B2) $$\frac{3}{4\pi }\rho ={\left(r+\delta \right)}^{3}-r^{3}\leftarrow \to 3r^{2}\delta +3r\delta^{2}+\left(\delta^{3}-\frac{3}{4}\frac{C}{\rho \pi }\right)=0$$

which is a second order equation in *r*.

So:

(B3) $$r=\delta \frac{\sqrt{\frac{C}{\rho \pi \delta^{3}}-\frac{1}{3}}-1}{2}$$

We can neglect the negative term; the other term is positive if:

(B4) $$\sqrt{\frac{C}{\rho \pi \delta^{3}}-\frac{1}{3}}-1>0\to C>\frac{4}{3}\rho \pi \delta^{3}$$

This condition indicates that there should be enough lipids to create a small sphere of radius δ (it is correct: otherwise, the membrane cannot be created …). So, the protocell internal volume V_r_ is:

(B5) $$V_{r}\left(C\right)=\frac{1}{6}\pi \delta^{3}{\left(\sqrt{\frac{C}{\rho \pi \delta^{3}}-\frac{1}{3}}-1\right)}^{3}$$

and, if δ ≪ 1, it can be approximated to:

(B6) $$V_{r}\left(C\right)\underset{\delta \to 0}{\approx }O\left(C^{\frac{3}{2}}\right)$$

As discussed in the text we assume that when C = θ, the protocell divides into two identical daughters whose volumes are θ/2 each. Therefore, if R is the internal radius of the parent protocell at division time, and r is the internal radius of each daughter protocell, one has:

(B7) $$\frac{\theta }{\rho }=\frac{4}{3}\pi {\left(R+\delta \right)}^{3}-\frac{4}{3}\pi R^{3}=2\left(\frac{4}{3}\pi {\left(r+\delta \right)}^{3}-\frac{4}{3}\pi r^{3}\right)$$

from the first equality one finds:

(B8) $$\frac{\theta }{\rho }=\frac{4}{3}\pi {\left(R+\delta \right)}^{3}-\frac{4}{3}\pi R^{3}\to 3R^{2}\delta +3R\delta^{2}+\delta^{3}-\frac{3}{4}\frac{\theta }{\rho \pi }=0$$

Then, solving the equation:

(B9) $$R=\delta \frac{\sqrt{\frac{\theta }{\rho \pi \delta^{3}}-\frac{1}{3}}-1}{2}$$

Similarly, for the daughter protocell one has:

(B10) $$r=\delta \frac{\sqrt{\frac{\theta }{2\rho \pi \delta^{3}}-\frac{1}{3}}-1}{2}$$

Therefore, the ratio between the daughter and the mother protocells’ volumes is:

(7) $$\frac{V_{r}\left(\theta /2\right)}{V_{r}\left(\theta \right)}=\frac{\frac{1}{6}\pi \delta^{3}{\left(\sqrt{\frac{\theta /2}{\rho \pi \delta^{3}}-\frac{1}{3}}-1\right)}^{3}}{\frac{1}{6}\pi \delta^{3}{\left(\sqrt{\frac{\theta }{\rho \pi \delta^{3}}-\frac{1}{3}}-1\right)}^{3}}\underset{\delta \to 0}{\approx }{\left(\sqrt{\frac{1}{2}}\right)}^{3}=0.3535\ldots$$

For a more general discussion of the division process see also [67].

## References

[1] Rasmussen S., Chen L., Deamer D., Krakauer D.C., Packard N.H., Stadler P.F., Bedau M.A. (2004). Transitions from nonliving to living matter. Science.

[2] Rasmussen S., Bedau M.A., Chen L., Deamer D., Krakauer D.C., Packard N.H., Stadler P.F. (2008). Protocells: Bridging Nonliving and Living Matter.

[3] Solé R.V., Munteanu A., Rodriguez-Caso C., Macía J. (2007). Synthetic protocell biology: From reproduction to computation. Philos. Trans. R. Soc. B: Biol. Sci..

[4] Carletti T., Serra R., Villani M., Poli I., Filisetti A. (2008). Sufficient conditions for emergent synchronization in protocell models. J. Theor. Biol..

[5] Filisetti A., Serra R., Carletti T., Villani M., Poli I. (2010). Non-linear protocell models: Synchronization and chaos. Eur. Phys. J. B.

[6] Ganti T. (2003). Chemoton Theory.

[7] Luisi P.L., Ferri F., Stano P. (2006). Approaches to semi-synthetic minimal cells: A review. Naturwissenschaften.

[8] Mansy S.S., Schrum J.P., Krishnamurthy M., Tobé S., Treco D.A., Szostak J.W. (2008). Template-directed synthesis of a genetic polymer in a model protocell. Nature.

[9] Morowitz H.J., Heinz B., Deamer D.W. (1988). The chemical logic of a minimum protocell. Orig. Life Evol. Biosph..

[10] Munteanu A., Solé R.V. (2006). Phenotypic diversity and chaos in a minimal cell model. J. Theor. Biol..

[11] Rasmussen S., Chen L., Nilsson M., Abe S. (2003). Bridging nonliving and living matter. Artif. Life.

[12] Rocheleau T., Rasmussen S., Nielsen P.E., Jacobi M.N. (2007). Emergence of protocellular growth laws. Philos. Trans. R. Soc. B: Biol. Sci..

[13] Segrè D., Lancet D. (2000). Composing life. EMBO Rep..

[14] Stano P., Luisi L.P. (2010). Achievements and open questions in the self-reproduction of vesicles and synthetic minimal cells. Chem. Commun..

[15] Szostak J.W., Bartel D.P., Luisi P.L. (2001). Synthesizing life. Nature.

[16] Hanczyc M.M., Szostak J.W. (2004). Replicating vesicles as models of primitive cell growth and division. Curr. Opin. Chem. Biol..

[17] Vasas V., Fernando C., Santos M., Kauffman S.A., Szathmary E. (2012). Evolution before genes. Biol. Direct.

[18] Solé R.V., Macía J., Fellermann H., Munteanu A., Sardanyés J., Valverde S., Rasmussen S., Bedau M.A., Chen L., Deamer D., Krakauer D.C., Packard N.H., Stadler P.F. (2008). Models of Protocell Replication. In Protocells: Bridging Nonliving and Living Matter.

[B19-life-04-00837] 19.This is of course an approximate description of the actual process of vesicle fission: the daughter cells may be of different size and, perhaps more important, the breakup is a complex process and it does not always take place at a fixed size. However, for modeling purposes, we will first consider the simplest version, and we will postpone to further works the consideration of fluctuations in the size of the daughter cells and in the value of the threshold on the parent cell volume that leads to fission

[20] Serra R., Carletti T., Poli I. (2007). Synchronization phenomena in surface-reaction models of protocells. Artif. Life.

[21] Wagner N., Ashkenasy G. (2009). Symmetry and order in systems chemistry. J. Chem. Phys..

[22] Sievers D., von Kiedrowski G. (1994). Self-replication of complementary nucleotide-based oligomers. Nature.

[23] Mansy S.S. (2009). Model protocells from single-chain lipids. Int. J. Mol. Sci..

[24] Segre D., Lancet D., Kedem O., Pilpel Y. (1988). Graded autocatalysis replication domain (GARD): Kinetic analysis of self-replication in mutually catalytic sets. Orig. Life Evol. Biosph..

[25] Kauffman S.A. (1986). Autocatalytic sets of proteins. J. Theor. Biol..

[26] Filisetti A., Graudenzi A., Serra R., Villani M., Fuchslin R.M., Packard N., Kauffman S.A., Poli I. (2011). A stochastic model of autocatalytic reaction networks. Theory Biosci..

[27] Carletti T., Filisetti A. (2012). The stochastic evolution of a protocell: The Gillespie algorithm in a dynamically varying volume. Comput. Math. Methods Med..

[28] Gillespie D.T. (1977). Exact stochastic simulation of coupled chemical reactions. J. Phys. Chem..

[29] Filisetti A., Graudenzi A., Damiani C., Villani M., Roberto S., Liò P., Miglino O., Nicosia G., Nolfi S., Pavone M. (2013). Role of Backward Reactions in a Stochastic Model of Catalytic Reaction Networks. Proceedings of the 12th European Conference on Artificial Life (ECAL2013).

[30] Filisetti A., Graudenzi A., Roberto S., Villani M., de Lucrezia D., Poli I., Lenaerts T., Giacobini M., Bersini H., Bourgine P., Dorigo M., Doursat R. (2011). Role of Energy in a Stochastic Model of the Emergence of Autocatalytic Sets. Proceedings of the 11th European Conference on Artificial Life (ECAL2011).

[31] Serra R., Filisetti A., Villani M., Graudenzi A., Damiani C., Panini T. (2014). A stochastic model of catalytic reaction networks in protocells. Nat. Comput..

[B32-life-04-00837] 32.In that paper we assumed that a semipermeable membrane exists, and that some chemical species can cross the membrane (leading to an instantaneous equilibrium between the internal and external chemical potential of those species) ,while for others crossing is prohibited

[B33-life-04-00837] 33.If R is the set of all possible reactions, a RAF set is defined as a subset R’ ⊆ R of reactions in which: (i) each reaction r ∈ R’ is catalyzed by at least one molecule type involved in R’ and (ii) all reactants and catalysts in R’ can be created from the species whose presence is guaranteed (the so-called food set F) by using reactions only from R’ itself. See Section 3 for more details

[34] Hordijk W., Steel M. (2004). Detecting autocatalytic, self-sustaining sets in chemical reaction systems. J. Theor. Biol..

[B35-life-04-00837] 35.The presence of a RAF set in this type of model of chemical reactions plays a role analogous to that of the presence of an eigenvalue with a positive real part in the case of linear kinetic equations discussed in [4]

[36] Markovitch O., Lancet D. (2014). Multispecies population dynamics of prebiotic compositional assemblies. J. Theor. Biol..

[B37-life-04-00837] 37.If not differently indicated in this article, all the kinetic constants of the same kind are set to the same value (see Appendix A for the details); note, however, that the structure of the relationships among RAFs, membranes and protocell growth does not depend on the particular values of these parameters (whereas, of course, these parameters determine the rates of the phenomena)

[38] Gillespie D.T. (1976). A General Method for Numerically Simulating the Stochastic Time Evolution of Coupled Chemical Reactions. J. Comput. Phys..

[39] Bagley R.J., Farmer J.D., Langton H.G., Taylor C., Farmer J.D., Rasmussen S. (1991). Spontaneous Emergence of a Metabolism. Artificial Life II.

[40] Stadler P.F., Schuster P. (1990). Dynamics of small autocatalytic reaction networks-I. Bifurcations, permanence and exclusion. Bull. Math. Biol..

[41] Stadler P.F. (1991). Dynamics of autocatalytic reaction networks IV: Inhomogeneous replicator networks. Biosystems.

[42] Stadler P.F., Schnabl W., Forst C., Schuster P. (1995). Dynamics of small autocatalytic reaction networks II replication, mutation and catalysis. Bull. Math. Biol..

[43] Jain S., Krishna S. (2001). A model for the emergence of cooperation, interdependence, and structure in evolving networks. Proc. Natl. Acad. Sci. USA.

[44] Hordijk W., Fontanari J.F. Catalytic Reaction Sets, Decay, and the Preservation of Information. Proceedings of the International Conference on Integration of Knowledge Intensive Multi-Agent Systems.

[45] Filisetti A., Graudenzi A., Serra R., Villani M., de Lucrezia D., Fuchslin R.M., Kauffman S.A., Packard N., Poli I. (2011). A stochastic model of the emergence of autocatalytic cycles. J. Syst. Chem..

[46] Adamala K., Szostak J.W. (2013). Competition between model protocells driven by an encapsulated catalyst. Nat. Chem..

[B47-life-04-00837] 47.The hypothesis that the two daughters have identical volumes is a non-essential assumption, because of the supposition that the division phenomenon happens at a given threshold independent from the initial size: conversely, it allows a more compact result presentation. In any case, we checked if the hypothesis can be released without effecting the presented results (simulations not shown)

[48] Farmer J.D., Kauffman S.A. (1986). Autocatalytic replication of polymers. Physica D.

[49] Hordijk W., Hein J., Steel M. (2010). Autocatalytic sets and the origin of life. Entropy.

[50] Dyson F.J. (1985). Origins of Life.

[51] Eigen M., Schuster P. (1977). A principle of natural self-organization. Naturwissenschaften.

[52] Jain S., Krishna S. (1988). Autocatalytic set and the growth of complexity in an evolutionary model. Phys. Rev. Lett..

[53] Heiner M., Gilbert D., Donaldson R., Bernardo M., Degano P., Zavattaro G. (2008). Petri Nets for Systems and Synthetic Biology in Formal Methods for Computational Systems Biology.

[54] Hordijk W., Steel M., Kauffman S.A. (2012). The structure of autocatalytic sets: Evolvability, enablement, and emergence. Acta Biotheor..

[B55-life-04-00837] 55.If R is the set of all possible reaction, a RAF set is defined as the subset R’⊆ R of reactions in which: (i) each reaction r ∈ R’ is catalyzed by at least one molecule type involved in R’ and (ii) all reactants and catalysts in R’ can be created from the molecules whose presence is guaranteed (the so-called food set F) by using reactions only from R’ itself. See [34] for a more detailed explanation

[B56-life-04-00837] 56.It should be observed that also periodic variations in the growth and duplication rates are compatible with a viable protocell population, provided that the variations are also synchronized (a phenomenon that was called supersynchronization in [5]

[57] Mavelli F., Ruiz-Mirazo K. (2013). Theoretical conditions for the stationary reproduction of model protocells. Integr. Biol..

[58] Plasson R., Brandenburg A., Jullien L., Bersini H. (2011). Autocatalysis: At the root of self-replication. Artif. Life.

[59] Kaneko K. (2006). Life: An Introduction to Complex Systems Biology.

[B60-life-04-00837] 60.Further novelties might be due to the interactions between different protocells, whose contents may differ (see Section 6 for a discussion on the role of heterogeneity in protocell populations). However, these novelties would be less radical than those discussed here; it has been argued that a system can be deemed truly evolvable only if it can accept some “true novelties” of the kind discussed in the text [17,27]

[B61-life-04-00837] 61.Or if it is capable to directly catalyze its own formation at a rate higher than those of the already existing ones; in this case the new irrRAF will replace the already existing ones

[62] Bird R.B., Stewart W.E., Lightfoot E.N. (1976). Transport Phenomena.

[63] Maynard Smith J. (1986). The Problems of Biology.

[64] Serra R., Villani M. (2013). Mechanism for the formation of density gradients through semipermeable membranes. Phys. Rev. E.

[65] Luisi P.L., Allegretti M., Souza T., Steineger F., Fahr A., Stano P. (2010). Spontaneous protein crowding in liposomes: A new vista for the origin of cellular metabolism. ChemBioChem.

[66] Maurer S.E., Monnard P.A. (2011). Primitive Membrane Formation, Characteristics and Roles in the Emergent Properties of a Protocell. Entropy.

[67] Carletti T., Fanelli D. (2007). From chemical reactions to evolution: Emergence of species. Eur. Phys. Lett..

